# *TP53* Expression and Mutational Analysis in Hematological Malignancy in Jeddah, Saudi Arabia

**DOI:** 10.3390/diagnostics12030724

**Published:** 2022-03-16

**Authors:** Heba Alkhatabi, Elrashed B. Yasin, Zeenat Mirza, Raed Alserihi, Raed Felimban, Aisha Elaimi, Manal Shaabad, Lina Alharbi, Hameeda Ahmed, Abdulrahman M. Alameer, Abdullah Ebraheem Mathkoor, Ahmed Salleh Barefah

**Affiliations:** 1 Department of Medical Laboratory Technology, Faculty of Applied Medical Sciences, King Abdulaziz University, Jeddah 21589, Saudi Arabia; halkhattabi@kau.edu.sa (H.A.); zmirza1@kau.edu.sa (Z.M.); aaalserihi@kau.edu.sa (R.A.); faraed@kau.edu.sa (R.F.); aelaimi@kau.edu.sa (A.E.); 2 Center of Excellence in Genomic Medicine Research (CEGMR), King Abdulaziz University, Jeddah 80200, Saudi Arabia; manalshaabad@gmail.com (M.S.); loo_oona@yahoo.com (L.A.); hameeda.ahmed@hotmail.com (H.A.); 3 Department of Medical Laboratory Technology, Faculty of Applied Medical Sciences, King Abdulaziz University, Rabigh 25732, Saudi Arabia; 4 King Fahd Medical Research Center, King Abdulaziz University, Jeddah 21589, Saudi Arabia; 5 Center of Innovation in Personalized Medicine (CIPM), King Abdulaziz University, Jeddah 21589, Saudi Arabia; 6 Sabya General Hospital, Jazan 82511, Saudi Arabia; a.m.alameer1986@gmail.com; 7 Jazan Armed Forces Hospital, Jazan 82511, Saudi Arabia; mathkoor2013@gmail.com; 8 Hematology Department, Faculty of Medicine, King Abdulaziz University, Jeddah 21589, Saudi Arabia; asbarefah@kau.edu.sa; 9 Hematology Research Unit, King Fahad Research Center, King Abdulaziz University, Jeddah 21589, Saudi Arabia

**Keywords:** *TP53* mutation, hematological malignancies, *TP53* deletion, myelodysplastic syndromes, FISH

## Abstract

Background: Tumor protein 53 (*TP53*) is a tumor-suppressor gene and plays an essential role in apoptosis, cell cycle arrest, genomic stability, and DNA repair. Although it is the most often mutated gene in human cancer, it has respectively low frequency in hematological malignancy but is significantly linked with complex karyotype, poor prognosis, and chemotherapeutic response. Nevertheless, the prevalence and prognostic role of *TP53* mutations in hematological malignancy in Saudi patients are not well reported. We, therefore, aim to assess the frequency of *TP53* mutations in hematological malignancies in Saudi Arabia. Method: 20 different hematological malignancy samples were tested using fluorescence in situ hybridization (FISH) technique for *TP53* deletion detection and next-generation sequencing (NGS) targeted panel was applied on 10 samples for mutations identification specifically *TP53* mutation. Results: *TP53* deletion was detected in 6 of 20 samples by FISH. Most of the 6 patients with *TP53* deletion had acute lymphoblastic leukemia (ALL), and majority of them were child. NGS result revealed one heterozygous missense mutation in exon 5 of the *TP53* gene (c. G9963A, p.H175R). Conclusion: To the best of our knowledge, the *TP53* mutation is novel variant, and the first time we are reporting their association with myelodysplastic syndromic individual with complex karyotype. This study recommends further analysis of genomic mutations on bigger cohorts, utilizing high throughput technologies.

## 1. Introduction

*TP53* is the most frequently mutated gene in most human cancers, with a frequency of 50% [1,2]. Alterations consist of mutations and deletions and are generally related to advanced disease stages, inadequate therapy response, and poor prognosis [3,4,5,6,7]. The transcription component *TP53* has a central regulatory role in numerous signaling pathways, including cell cycle arrest, apoptosis, and DNA repair [8,9]. Owing to its essential function of maintaining genome stability, the p53 protein has been described as ‘the guardian of the genome’. *TP53* deletions are frequently observed to be related to *TP53* mutations of the second allele, assisting the ‘two-hit’ hypothesis, which indicates that alteration of each copy of a tumor suppressor gene is required to result in and/or force most cancers development [2,10,11,12,13]. p53 activation takes place in response to DNA damage or different stress conditions (for instance, metabolic changes, hypoxia, or oncogene activation), leading to activation or repression of its target genes, precisely inflicting G1 cell cycle arrest and apoptosis induction, a procedure this is disrupted with the aid of using *TP53* mutation/deletion in cancer [14,15,16]. Deletions in *TP53* often result from large deletions of the short arm of chromosome 17, wherein *TP53* is located, which may be detected through interphase FISH (fluorescence in situ hybridization), figuring out the copy-number state of a gene. Thus, the *TP53* feature is commonly preserved within a *TP53* deletion without accompanying *TP53* mutation within the other allele. Mutations in *TP53* generally bring about a lack of character of the p53 protein that could encompass complete or partial absence of characteristic, depending on the site of the mutation [17]. Whereas tumor suppressors are usually inactivated through frameshift or nonsense mutations, the most common mutation form of *TP53* in tumors is represented through missense mutations within the coding region [10,18]. Although the cancer-related *TP53* mutations are determined at various positions throughout the *TP53* sequence, they generally cluster within the DNA-binding domain, disrupting the ability of p53 to bind to its target DNA sequences, therefore preventing transcriptional activation of the respective genes [19]. About 30% of the missense mutations are located in six ‘hotspot’ residues (p.R175, p.G245, p.R248, p.R249, p.R273, and p.R282) withinside the DNA-binding domain of p53, with R273 and R248 being the most often mutated ones [20,21]. Interestingly, even though *TP53* mutations usually abolish the tumor suppressor activity of the protein (loss-of-function mutations), gain-of-function mutations have additionally been defined that cause acquisition of additional oncogenic functions that promote cell growth and provide survival advantages to the cell [21].

Despite the huge diversity in the genes implicated in tumorigenesis, the *TP53* mutations is most frequently associated with poor prognostic outcome in all type of cancer. However, *TP53* mutations were reported to occur in almost every type of cancer and less frequent in hematological malignancies [1]. Mutations and deletions in *TP53* are determined in all hematological malignancies at varied frequencies. Whereas *TP53* mutations had been determined to arise pretty frequently in ALL (16%) [22] and AML (12%) [23,24], the frequencies are decreased in CLL (7%) [7,25,26,27] and MDS (6%) [28,29,30]. Like most cancer types, *TP53* mutations in hematological malignancies had been determined to expose a negative effect on survival (23–30). Moreover, *TP53* mutations had been proven to be enriched in therapy-related diseases such as t-AML and t-MDS. They were also determined excessively in relapse cases, which were related to poor outcomes [31,32]. Therefore, the proposed role of *TP53* mutations in therapy-associated patients and relapsed disease appears to be because of the selective gain of the individual cells due to their resistance to therapies [10,33].

Cancer epidemiology in Saudi Arabia (SA) differs from that of the USA with respect to types of common malignancies, some cancers magnitude 3-fold in the latest years. This increases can be attributed to genetic factors in addition to other factors as SA carries one of the highest rates of consanguinity worldwide [1]. Hematologic malignancies are among the top five cancers prevalent in SA, including lymphoma and leukemia. According to the reported data from the GLOBOCAN for region of Middle-East and Northern Africa (MENA), the estimated crude incidence is 5.3 per 100.000 among male population and 4.0 per 100,000 females. Moreover, Gulf Cooperation Council report on cancer, ranked leukemia as the 4th among the most common cancers in the area. The Saudi Cancer Registry, stated that leukemia was ranked 5th among cancers in both genders of all ages in the Saudi population [2]. Currently, no sufficient data exist on *TP53* in hematological malignancies in Saudi Arabia. Therefore, we aimed to study the frequency and the type of mutations associated with *TP53* in hematological malignancies. We carried out a comprehensive analysis of the *TP53* gene in different hematological malignancies, such as AML, MDS, and ALL. The analysis include: (i) frequency assessment of *TP53* mutations and larges deletions using different technologyies, (ii) discovering the types of mutation, (iii) identifying the correlations to cytogenetic aberrations, and (iv) characterizing the age dependency.

## 2. Materials and Methods

### 2.1. Patients

We recruited 20 cases of hematological malignancies including nine AML (4 females and 5 males), nine ALL (4 females and 5 males), one myelodysplastic syndromic (female), and one non-Hodgkin’s lymphoma (female) with a median age of 43 years (range: 2–69 years). All selected samples were collected from patients at diagnostic stage (before treatment.) Bone marrow specimens were collected from patients at King Abdulaziz University Hospital during the year 2015–2017. Ethical approval was obtained from the local ethical committee (Bioethical approval code: 01-CEGMR-Bioeth-2019) and the rules of the Helsinki Declaration were followed in the study.

### 2.2. Cytogenetic and FISH Analysis

Chromosomal analysis using G-banding was conducted for recruited cases according to standard protocol [34,35,36]. ISCN guidelines (2016) were followed for the nomenclature of karyotypes [37]. Further, to determine the copy-number state of *TP53* in patients, interphase FISH using Vysis probes for *TP53* spanning a 167 kb region in 17p13, including the complete sequence of *TP53*, was performed including preparation of the interphase/metaphase spreads, denaturation of the target DNA, DNA probing, hybridization, washing, and counterstaining. Signal and image analysis were done using Axioplan 2 and Axioskop 2 imaging fluorescence microscope (Carl-Zeiss, Göttingen, Germany). Signals were counted for complete metaphase and non-overlapped interphase cell within the chromosome and nuclear boundary until 200 metaphase and interphase nuclei were enumerated and analyzed. In normal cells, two green signals (control probe for 17 centromere, D17Z1) and two red signals (P53, 17p13.1, probe) were observed.

### 2.3. Next-Generation Sequencing Analysis

To detect the variants in P53 and other target genes, panel sequencing analysis was performed for 10 selected cases, according to the variability in disease diagnosis (AML, ALL, MDS, and NHL) and chromosomal abnormalities detected by karyotype and FISH. ClearSeq AML HS panel (G9963A, Agilent Technologies, Santa Clara, CA, USA), targeting 48 exons among 20 myeloid leukemia-associated genes, was used to investigate the mutational hotspot regions of *TP53* (ENST00000269305, exons 5–8) and other panel genes (Table 1). Genomic DNA was extracted from the patient’s bone marrow using QIAamp DNA blood Mini kit (51,104, QIAGEN, Hilden, Germany) as per the manufacturer’s instructions and quality was assessed by a NanoDrop2000c (5538, Thermo Scientific, Waltham, MA, USA). Purity was determined by absorbance ratio (A260/A280 = 1.7–1.9). DNA was digested and denatured to generate different fragments or target regions using the HaloPlex HS Target Enrichment System kit, (G9963A, Agilent Technologies, Santa Clara, CA, USA). Fragmented target DNA was hybridized with a library probe (HaloPlex HS probes), followed by streptavidin ligation and barcode target capturing and amplification of enriched fragment. The template library was denatured and diluted to 20 pM before next-generation sequencing using MiSeq (Illumina, San Diego, CA, USA) platform and ClearSeq AML HS panel according to manufacturer’s protocol.

### 2.4. Data Analysis

Data acquisition and analysis were performed using Agilent’s SureCall V2. (Agilent Technologies, Santa Clara, CA, USA) that incorporates BWA, SAM tools (Agilent Technologies) for alignment, variant calling, and annotation. Validity of the somatic mutations was checked using COSMIC v74 database (http://cancer.sanger.ac.uk/cancergenome/projects/cosmic, accessed on 25 January 2022) and functional interpretation was performed using SIFT 1.03 (http://sift.jcvi.org, accessed on 25 January 2022) and PolyPhen 2.0 (http://genetics.bwh.harvard.edu/pph2, accessed on 25 January 2022) tools. Furthermore, *TP53* variants were verified using the IARC repository (r17).42. Single-nucleotide polymorphisms (SNP) were annotated according to the NCBI dbSNP (http://www.ncbi.nlm.nih.gov/snp, accessed on 25 January 2022; Build 144) database. Synonymous variants and alterations within introns were not scored except for splice-site mutations at position ± 1 or 2. Missense variants, which did not have unique entries in the COSMIC or dbSNP databases, were annotated as variants of unknown significance (VUS).

### 2.5. Structural Analysis

Expasy’s uniport database was searched for each human DNA-binding protein; p53 (P04637), ASXL1 (Q8IXJ9-1), and SETBP1 (Q9Y6X0). The Protein Data Bank (PDB) of Research Collaboratory for Structural Bioinformatics (http://www.rcsb.org/, accessed on 25 January 2022) was searched and three-dimensional structure of human *TP53* (PDB code: 2PCX) was retrieved. There weren’t any experimentally predetermined structures for ASXL1 and SETBP1 in PDB, so AlphaFold predicted model AF-Q8IXJ9-F1 and AF-Q9Y6X0-F1 respectively. Impact of specific mutation on structures were visualized and site-specific mutagenesis was done using Schrodinger’s PyMOL.

## 3. Results

### 3.1. Clinical Characteristics of Patients

A total of 20 hematological malignancies patients (10 males and 10 females) with a median age of 43 years (range 5–69 years) were included in present study (Table 2). Most cases were of AML (45%) and ALL (45%) categories followed by non-Hodgkin’s lymphoma (5%) and MDS (5%).

### 3.2. Cytogenetic and FISH Results

**Figure 1:**
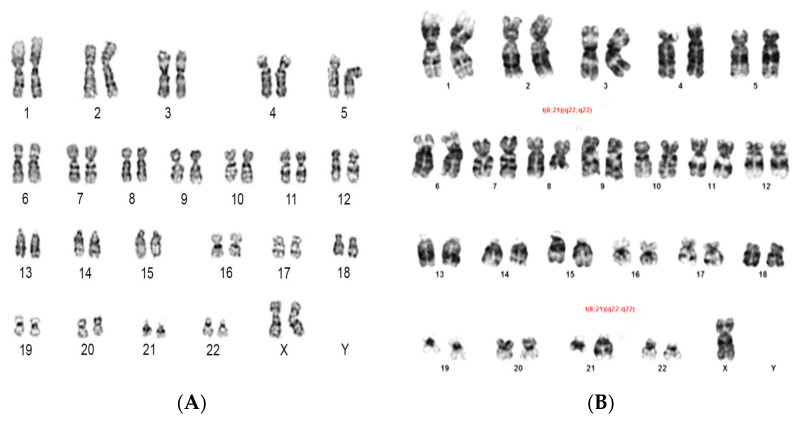

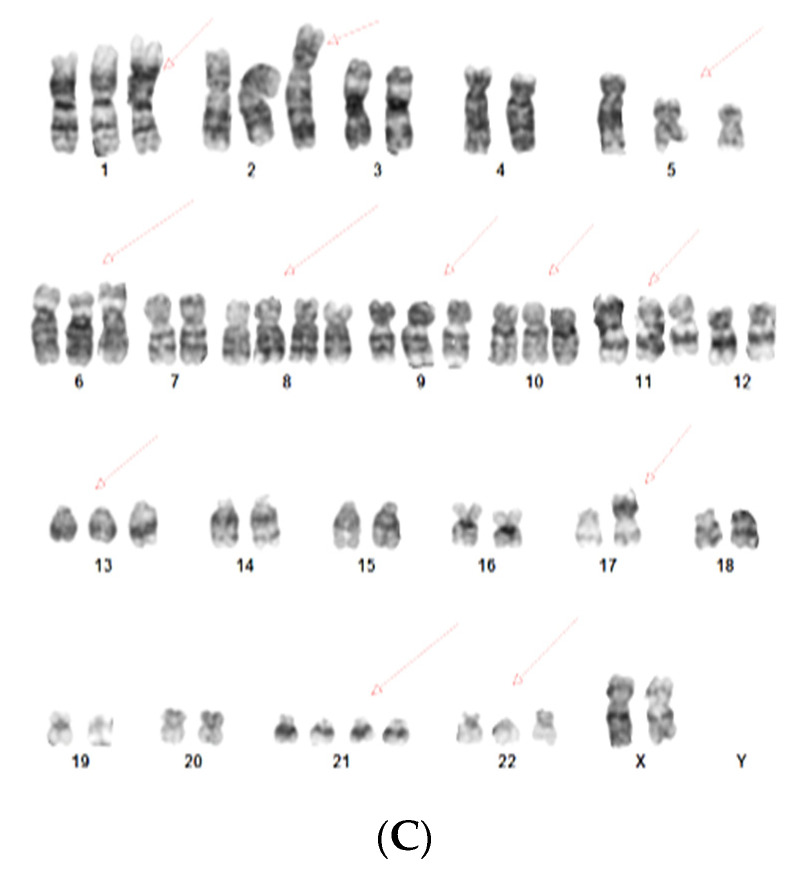
Demonstrated cytogenetic result. (**A**) represented AML female with normal karyotype. (**B**) ALL patient with single chromosome abnormality, 46, XY, t (8; 21) (q22; q22). (**C**) MDS patient with complex karyotype, 65-58, XX, +1, +2, der (2) t (2;5) (q12; q37), +5, +6, +8, +9, +10, +11, der (17) t (12;17) (p10; p10), +13, del (13) (q21), +21, +21 [cp50]).

**Figure 2:**
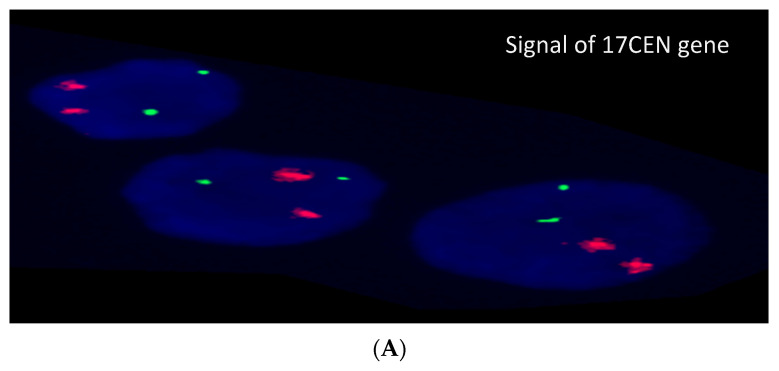

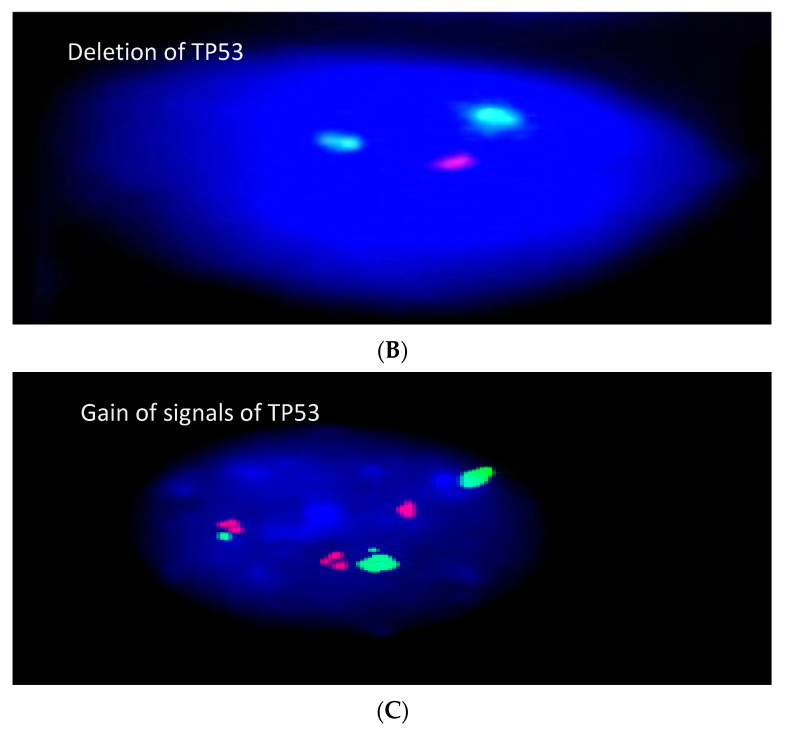
Result of *FISH* analysis. (**A**) represents a normal result (2 green and 2 red signals). (**B**) *TP53* deletion (2 green and 1 red signals). (**C**) represents cases with trisomy singles (3 green and 3 red).

The cytogenetic results showed complex karyotype in four cases (20%) and single chromosomal abnormality in two cases (10%) while no chromosomal abnormalities were detected in remaining cases (70%) (Table 2, Figure 1) FISH results, based on the analysis of 200 interphase cells, were variable as normal signaling for *TP53* found in seven, partial deletion for *TP53* (11–45% of interphase cells) found in seven, mixed signaling of *TP53* (15% normal cells, 45% cells with deletion in *TP53*, and 40% cells had three signals for *TP53*) found in one case and extra signals for *TP53* (three signals in 20 and 30% without deletion of *TP53*) were present in two cases (Figure 2). The *TP53* deletion was detected in five ALL (55%), one AML and one MDS patient while *TP53* amplification was detected in one NHL case only. There was no gender association with the *TP53* deletion. However, *TP53* deletions were detected in 62% (5/8) child samples compared to 16% (2/12) adult patient (Table 3).

### 3.3. Next-Generation Sequencing Analysis

Sequencing analysis revealed a heterozygous missense mutation (c. G9963A, p.H175R) in the *TP53* gene in MDS patient where substitution of T to C resulted in a change of amino acid from histidine to arginine at codon 175 (Table 4).

### 3.4. Correlation of TP53 Mutation with Cytogenetic & FISH Results

In assessing the relationship of *TP53* mutation to cytogenetic and FISH results, the mutation was observed in MDS patients with a complex karyotype. Interestingly, the FISH result for this patient showed a gain of *TP53* gene in 60 cells out of 200 investigated cells.

### 3.5. Correlation of TP53 Mutation with Other Genes Mutations

**Figure 3:**
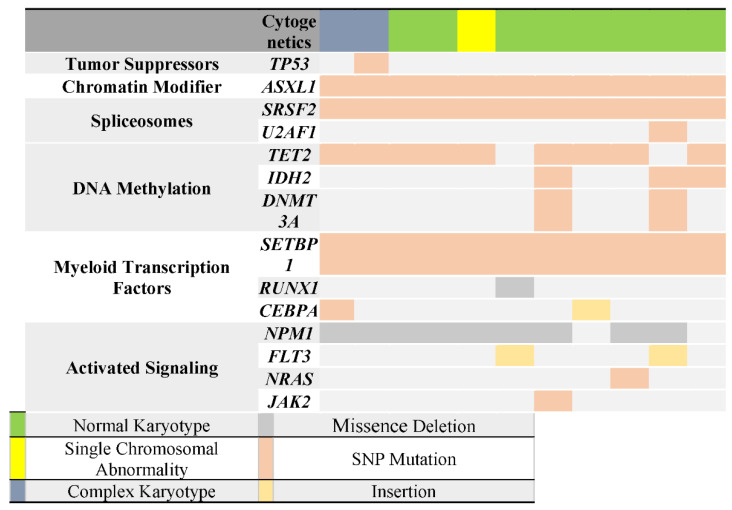
Mutation status according to patient characteristics & cytogenetics. The far-left column lists the 14 genes that were tested in the panel. Each column represents a single patient, and each colored bar indicates the presence of a mutation in the indicated gene. In addition, each color represents the type of mutation and cytogenetic status, as shown above. This illustrates the spectrum of coexistent mutations in all patients.

Using targeted NGS with ClearSeq AML HS panel (Agilent Technologies), we identified a total of 91 mutations in 14 of the 20 genes analyzed in our cohort. The analysis showed that patient with *TP53* mutation also had mutations in *NPM1*, *TET2*, *SRSF2*, *ASXL1*, *SETBP1* (Table 5). Furthermore, among these mutations, there were two unique mutations in both *ASXL1* (K1368T) and *SETBP1* (V231L) that were exclusively associated with *TP53* mutation and did not present in the other patients (see Table 6, Figure 3).

### 3.6. Structural and Functional Impact at the Protein Level

**Figure 4:**
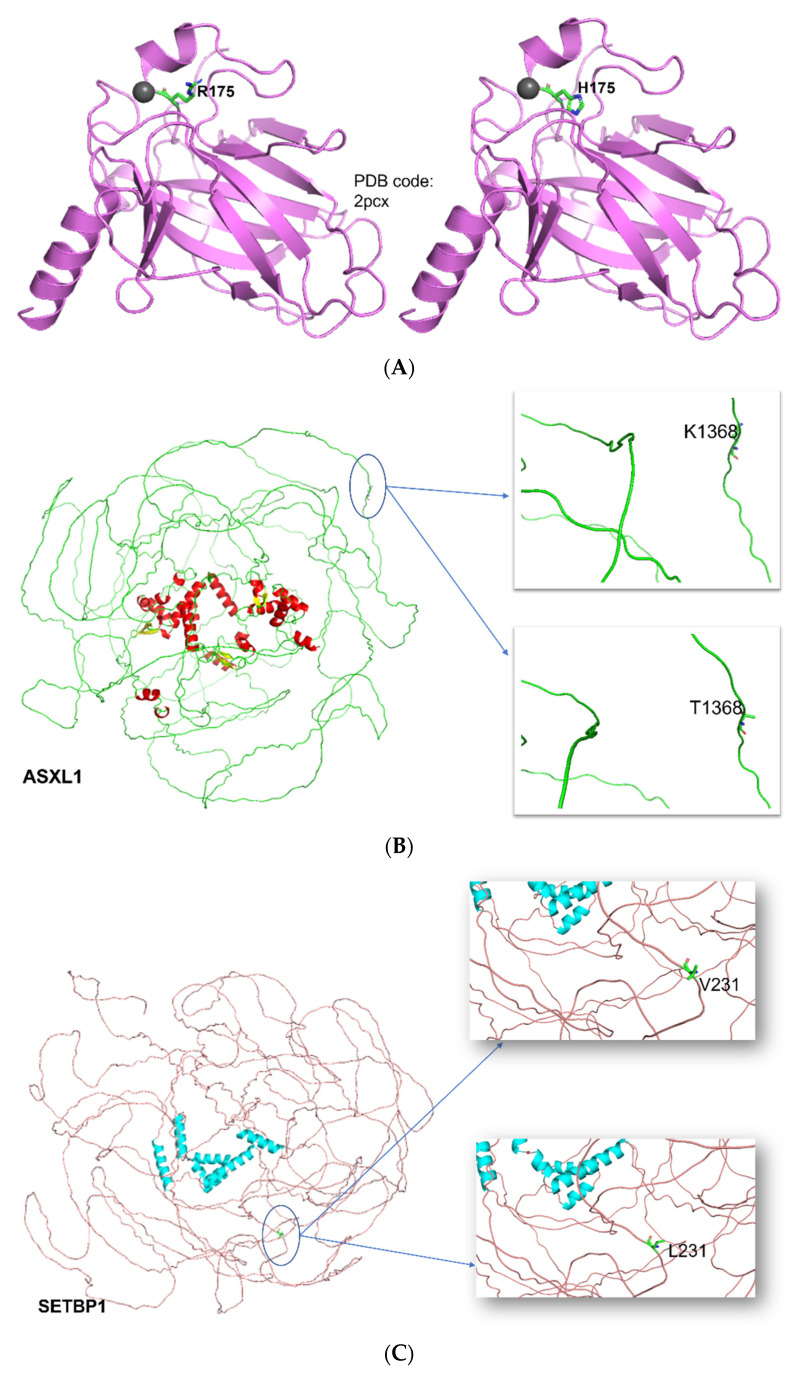
Structural reflection of the mutations. (**A**) Three-dimensional structure of p53 showing wild (R175) and mutated (H175); zinc atom shown as grey sphere. (**B**) Three-dimensional structure of ASXL1 showing wild (K1368) and mutated (T1368). (**C**) Three-dimensional structure of *SETBP1* showing wild (V231) and mutated (L231).

**Figure 5:**
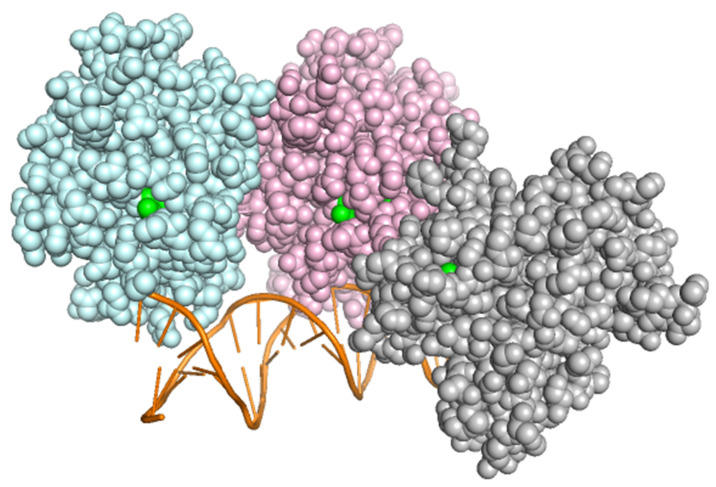
p53 interaction with DNA helix (orange), 3 subunits shown (colored differently), Arg175 shown in green.

Three-dimensional structures of *TP53*, *ASXL1* and *SETBP1* were visualized, and the changes induced by specific mutations were focused (Figure 4). In p53, position Arg175 is one of the hot-spots for mutation in human cancer [38], because this residue plays an important role in maintaining the structure of the DNA-binding domain but isn’t involved in direct interaction with DNA (Figure 5). Arginine to histidine mutation might executes its function by directly binding other transcription factors and gene promoters and transcriptionally altering their expressions by recruiting cofactors or corepressors. The mutations found in *ASXL1* and *SETBP1* are located on the flexible loop and are on the periphery, they might be altering their interactions with other proteins (Table 7).

## 4. Discussion

*TP53* is a major tumor suppressor which plays an important role in tumorigenesis, proliferation, and cell survival in most human cancers [39]. The previous research confirmed that greater than 80% of human cancers have mutations in *TP53*. The current model of the IARC database (R20, July 2019) includes over 29,900 somatic mutations and 9200 variations reported in SNP databases (“Database Development”, 2019). Nowadays, it is undisputed that the inactivation of the *TP53* gene due to a mutation is a critical step in tumor transformation and progression [1]. The activity of *TP53* lies in its ability to activate and suppress a broad set of target genes whose products regulate, among other things: the cell cycle arrest and apoptosis when the DNA is damaged [40].

*TP53* gene might not have a specific role in developing all tumors. However, mutations of this gene have been related to a complicated karyotype, poor prognosis, and poor response to chemotherapy [41,42,43]. There is a lack of the published data in Saudi Arabia that describe the frequency of the *TP53* mutations and their relationship with cytogenetic and clinical phenotype in hematological neoplasms. Therefore, we endeavored in this study to evaluate the *TP53* deletion using the FISH technique and *TP53* mutations screening using NGS technology and their relationship with cytogenetics and clinical phenotype in leukemia patients.

Previous studies have shown that FISH is a powerful cytogenetic technique used to evaluate the *TP53* alterations in patients with hematological malignancies [44,45]. In our study, 20 patients’ samples were examined, and the *TP53* deletion was detected in 35% of the cases. Similarly, there were about 35% of cases with normal signaling of *TP53* and two cases with extra signals in *TP53*. This study showed that, *TP53* deletion was identified in about 62.5% of all investigated child samples, whereas the deletion was detected at a lower rate (16%) of adult cases. Furthermore, the highest average of *TP53* deletion has been noticed in patients with ALL (55%), which is in concordance with and even higher than what was reported by other studies (56%) [46]. *TP53* changes were mainly seen in a hypodiploid subtype of ALL, mainly due to germline changes, which changed the disease manifestation to Li-Fraumeni syndrome. Therefore, it becomes important to know if the identified variant is a secondary event contributing to risk stratification and treatment response [47].

Gain of mutation for *TP53* was observed in two cases; one of them was an adult male patient with AML and had a normal karyotype. The other one was an adult female with MDS and with complex karyotype. The MDS patient had an abnormality on chromosome 17, and that distribution in the chromosome structure might be associated with the extra signals detected by FISH. Therefore, to evaluate if the detected changes by FISH were originally derived from a mutation on the *TP53* gene or whether the gene is intact in the positive cases by FISH, NGS sequencing using a targeted panel was performed on 10 selected samples [47].

According to the analysis of 10 samples by NGS, only one (MDS patient) was harboring a *TP53* mutation in exon 5. The detected mutation was a heterozygote point mutation (T to C) that changed amino acid residue from histidine to arginine at codon 175 of the *TP53* gene. The mutation was found in an MDS patient who was the only case in the study. Based on our knowledge and from the search on different databases (ClinVar-NCBI”, 2020; “IARC *TP53* Search”, 2020; “Search results on cosmic for H175R”, 2020), this particular mutation (H175R) we observed in our study was not reported previously in MDS or any other hematological malignancies. However, this mutation was found in lung adenocarcinoma from Korean patients [48]. According to cytogenetic and FISH results, the mutation was associated with a complex karyotype and *TP53* gene amplification detected by FISH. This finding aligns with what was published before that *TP53* mutation is associated with a complex karyotype and poor prognosis in MDS [49,50].

p53 tumor suppressor homotetramer structure is composed of four identical protein chains tied together by the tetramerization domain at the center. Zinc is an essential cofactor with 1 zinc ion per subunit. A long flexible region in each chain then connects to the second stable domain: a large DNA-binding domain, rich in arginine residues that recognizes DNA’s specific regulatory sites and interacts with DNA. The transactivation domain found near the end of each arm, activates the neighboring proteins involved in DNA-reading machinery. R175H is a hotspot mutation (corresponds to variant dbSNP:rs28934578). This missense variant found in Li-Fraumeni syndrome (LFS), germline mutation, in sporadic cancers and somatic mutation. This natural SNV found in the DNA-binding domain, involved in positioning other DNA-binding amino acids. Arg175 belongs to region required for interaction with HIPK1, ZNF385A, FBXO42 and AXIN1. It does not induce SNAI1 degradation but reduces interaction with ZNF385A and causes loss of susceptibility to calpain.

The reason why *TP53* mutations are associated with the complex karyotype remains unclear and raises the question of whether these mutations promote and induce increasing cellular instability or whether these mutations are secondary mutations that occur only after chromosomal instability. Previous studies showed that *TP53* mutations in hematological malignancies are highly prevalent in a complex karyotype and deletion of chromosome 17p. At the same time, in the other cytogenetic subgroups, they are deficient, suggesting that chromosomes instability may precede mutations in *TP53* [22,51,52]. However, further studies and examination on larger cohorts are needed to assess these possibilities.

Targeted NGS in our research enabled us to discover mutations in other genes rather than *TP53*. The analysis revealed that *TP53* mutation was associated with other genes mutations such as *TET2*, *SRSF2*, *ASXL1*, *U2AF1*, *NPM1*, and *SETBP1*. Similar co-occurrence results for these mutations with *TP53* mutation in MDS were published [53,54,55] Interestingly, among these mutations, we found exclusive mutations on *ASXL1* (K1368T) and *SETBP1* (V231L) that were associated mainly with *TP53* mutation [56]. Reported that *ASXL* mutations are frequently seen in MDS in association with *SETBP1* mutations, inhibiting myeloid differentiation and inducing leukemic transformation [57]. Furthermore, they reported that *SETBP1* is a driver for *ASXL1* mutation, and *ASXL1* is a poor prognostic biomarker associated with short survival. Another study focused on *TP53* and *ASXL1* prognosis in AML and MDS reported that they are two independents factors associated with poor prognosis and short survival; nevertheless, none of the studies had reported the pathogenic significance of the particularly identified mutations on these genes, their importance on disease pathogenicity cannot be ignored and further functional validation should be done [58].

*ASXL1* codes for Polycomb group protein *ASXL1*, a huge 165.432 kD and length of 1541 amino acid residues. Variant K1368T isn’t yet reported. As there were no experimentally determined structures, AI-based predicted Alphafold structure was used. Residue of interest 1368 is three-dimensionally located on the flexible loop at periphery. Based on our knowledge, mutation of ASXL1 (K1368T) was also not previously reported, and its pathogenicity was not assessed or examined before. On the other hand, a *SETBP1* (V231L) mutation was found in Schinzel-Giedion Midface Retraction Syndrome with a mild effect, as reported by Illumina Clinical Services Laboratory (“VCV000159885.1-ClinVar-NCBI”, 2020). *SETBP1* (SET Binding Protein 1) doesn’t have any experimentally determined structures, hence, AI-based predicted Alphafold structure was used. It seems to have disordered regions. SNV V231L (corresponds to variant dbSNP:rs11082414) is benign as per ClinVar. It is a DNA-binding protein, functioning as an epigenetic hub that joins group of proteins that act together on histone methylation to make chromatin more accessible and regulate gene expression (Piazza et al., 2018). Not much is known about the overall function of the *SETBP1* protein and the effect of SET binding.

Therefore, the exclusiveness of the identified mutations in this project will be considered variants with unknown significance. As for the correlation of *TP53* mutations with tumor type and cytogenetic abnormalities, in AML, all patients were found with wild-type *TP53* (six patients had a normal karyotype and one with a single chromosomal abnormality). In addition, one patient has *TP53* deletion by FISH. This finding is consistent with other published work, which indicated that *TP53* mutations are infrequent in AML without a complex karyotype, highlighting its importance as a therapeutic target through activation of the intact gene [51,59,60].

In the lymphoma patient, there was no *TP53* mutation. Instead, the patient had a normal karyotype with a *TP53* deletion based on FISH. This finding is consistent with study of Ahmad et al., which revealed that *TP53* mutations in Saudi non-Hodgkin’s lymphoma are infrequent, as, from 45 patients, only one patient showed a mutation in the *TP53* gene [61]. For ALL, only one case was selected for NGS analysis for a patient with a complex karyotype and *TP53* deletion according to the FISH result, and no mutation was detected in *TP53*. Although in a 2014 study, Stengel et al. revealed that *TP53* mutations were above average in ALL with complex karyotype, the patient did not show any mutation in *TP53* [22].

## 5. Conclusions

Further examination and screening on a larger cohort is highly recommended to confirm our research findings. Also, the used panel covers only 4 exons from *TP53*, representing the exons that include the most reported hotspot mutations in the gene. This limits the study finding as there might be a chance of detecting other variants of the *TP53* gene on the uncovered regions. Therefore, whole gene sequencing for *TP53* is important to confirm the absence of any changes on the gene to support the recommendation of utilizing the activation of the wild-type gene in controlling tumor progression. Moreover, the FISH technique remains a powerful tool for clinical diagnosis, and further screening on the clinical impact of FISH analysis for *TP53* on AML and ALL manifestation is recommended.

## Figures and Tables

**Table 1:** ClearSeq AML HS panel.

Gene List (Targeted Exons)							
GENE	EXON	GENE	EXON	GENE	EXON	GENE	EXON
*ASXL1*	12	*EZH2*	8, 17, 18	*MPL*	10	*SF3B1*	13–15, 17
*CSF3R*	14, 17	*FLT3*	14, 20	*NPM1*	11	*SRSF2*	1
*CBL*	8, 9	*IDH1*	4	*NRAS*	2, 3	*TET2*	3, 9, 10, 11
*FFCEBPA*	1	*IDH2*	4	*RUNX1*	3, 4, 8	*TP53*	5–8
*DNMT3A*	4, 8, 13, 15, 16, 18–23	*JAK2*	12, 14	*SETBP1*	3	*U2AF1*	2, 6

**Table 2:** Patient information and cytogenetic result.

Case N.O.	Age	Sex	Diagnosis	Cytogenetic Result
1	2	M	ALL	46, XY
2	2	M	ALL	AL,57~43, XY, +X, dup (1) (q21q31), +4, +5, +6, +7, −8, +9, +10, +14, +17, +18, −19, −20, +21, +22 [cp50]
3	5	F	ALL	Leukemia,46, XX, der (19) t (1;19) (q25; p13.3) [17]/46, XX, idem, +der (21) t (1;21) (p13; p11.2) [14]/46, XX [19]
4	5	M	ALL	46, XX [20]
5	7	F	ALL	46, XX [20]
6	9	M	AML	46, XY [20]
7	12	M	ALL	46, XY, t (8; 21)(q22; q22) [29]/46, XY [21]
8	31	M	ALL	46, XY [20]
9	36	F	AML	46, XX [20]
10	43	M	AML	46, XY [20]
11	44	F	ALL	ALL,45, XX, +X, −9, t (9;22) (q34; q11.2), −13[cp34]/45, XX, t (9;22) (q34; q11.2) [cp8]/46, XX[cp8]
12	45	F	ALL	46, XX [20]
13	59	F	Lymphoma, NHL	46, XX [20]
14	63	F	AML	46, XX [20]
15	65	F	MDS	65-58, XX, +1, +2, der(2) t(2;5) (q12; q37), +5, +6, +8, +9, +10, +11, der(17) t (12;17) (p10; p10), +13, del (13) (q21), +21, +21 [cp50]
16	72	M	AML	45, XY,der (7;12), (q11.2; p12) [30]
17	13 Y	F	AML	46, XX [20]
18	35 Y	F	AML	46, XY [20]
19	42 Y	M	AML	46, XX [20]
20	69 Y	M	AML	46, XY [20]

**Table 3:** FISH result in relation with the clinical diagnosis and cytogenetic finding.

Diagnosis	Cytogenetic	FISH
NHL (1), ALL (5), AML (8)	Normal karyotype (14)	NHL *TP53* deletion (1/1) (11%)
		ALL *TP53* deletion (2/5) (20–22%)
		AML *TP53* deletion (1/8) (11%)
		AML *TP53* trisomy (1/8) (20%)
ALL (1), AML (1)	Single abnormality (2)	ALL *TP53* deletion (1/1) (22%)
		AML *TP53* deletion (0/1)
MDS (1), ALL (3)	Complex karyotype (4)	MDS (1/1) *TP53* trisomy (30%)
		ALL (2/3) *TP53* deletion (45%)

**Table 4:** NGS results for MDS sample (shows the mutation details).

Impacted Gene	*TP53*
Type of Mutation	Missense mutation (Heterozygous)
Chromosome	17
Ref. Allele	T
Alt. Allele	C
Function Class	Missense
AA	H175R
Codon	cAt/cGt
Quality	Pass
Allele Frequency	0.447
Number of Variant Alleles	10,232
Filtered Read Depth (per sample)	22,870
Effect	UNKNOWN
Exon ID	NM_001126118.ex.5

**Table 5:** Distribution Pattern of Coexisting Mutations in Patients with and without *TP53* Mutations.

Gene	Total No. of Mutation (*n* = 10)	*TP53*-Mutated (*n* = 1)	*TP53*-wt (*n* = 9)
*ASXL1*	10	1	9
*CEBPA*	2	0	2
*DNMT3A*	2	0	2
*FLT3*	2	0	2
*IDH2*	3	0	3
*JAK2*	1	0	1
*NPM1*	8	1	7
*RUNX1*	4	0	4
*SETBP1*	10	1	9
*SRSF2*	10	1	9
*TET2*	8	1	7
*U2AF1*	1	0	1
*NRAS*	1	0	1

**Table 6:** *ASXL1* & *SETBP1* mutations in all cases. The mutations marked with red color represent the exclusive association with *TP53* mutation.

Sample No.	Age	Sex	Diagnosis	Gene	Type of Mutation
1	5 Y	F	ALL	*ASXL1*	Missense (L815P)
				*SETBP1*	Silent (S1275)
2	65 Y	F	MDS	*ASXL1*	Missense (L815P) Missense (K1368T)
				*SETBP1*	Missense (V231L) Silent (S1275)
3	59 Y	F	Lymphoma, NHL	*ASXL1*	Missense (L815P) Silent (S1253)
				*SETBP1*	Missense (V1101I) Silent (S1275)
4	63 Y	F	AML	*ASXL1*	Missense (L815P)
				*SETBP1*	Silent (S1275)
5	36 Y	F	AML	*ASXL1*	Missense (L815P) Silent (S1253)
				*SETBP1*	Silent (H1206) Silent (S1275) Silent (L1278)
6	43 Y	M	AML	*ASXL1*	Missense (L815P) Silent (S1253)
				*SETBP1*	Silent (S1275)
7	13 Y	F	AML	*ASXL1*	Missense (L815P) Silent (S1253)
				*SETBP1*	Silent (H1206) Silent (S1275)
8	35 Y	F	AML	*ASXL1*	Missense (L815P) Silent (S1253)
				*SETBP1*	Missense (V1101I) Silent (S1275)
9	42 Y	M	AML	*ASXL1*	Missense (L815P) Silent (S1253)
				*SETBP1*	Silent (S1275)
10	69 Y	M	AML	*ASXL1*	Missense (L815P)
				*SETBP1*	Silent (S1275)

**Table 7:** Prediction of impact of mutations.

Gene-Mutation	Polyphen-2	I-Mutant 2.0	ClinVar
*TP53*-R175H	POSSIBLY DAMAGING score: 0.881 (sensitivity: 0.82; specificity: 0.94)	Decrease in stability ΔΔG = −1.35 Kcal/mol	PATHOGENIC
*ASXL1*-K1368T	BENIGN score: 0.091 (sensitivity: 0.93; specificity: 0.85)	Decrease in stability ΔΔG = −0.63 Kcal/mol	-
*SETBP1*-V231L	BENIGN score: 0.006 (sensitivity: 0.97; specificity: 0.75)	Decrease in stability ΔΔG = −0.19 Kcal/mol	BENIGN

## Data Availability

The data presented in this study are available on request from the corresponding author.
